# No effects of four weeks of combined brace and Schroth therapy in moderate adolescent idiopathic scoliosis on plantar pressure distribution

**DOI:** 10.1186/s12891-024-07841-z

**Published:** 2024-09-11

**Authors:** Daniela Ohlendorf, Christian Schlegel, Gerhard M. Oremek, Fabian Holzgreve, Eileen M. Wanke, Christian Mauer-Grubinger, Panagiotis Diaremes, Christian Hülstrunk, Omar Zabar, David A. Groneberg

**Affiliations:** 1 Institute of Occupational, Social and Environmental Medicine, Goethe University Frankfurt/Main, Theodor-Stern-Kai 7, Building 9a, 60596 Frankfurt/Main, Germany; 2 Clinic for Trauma Surgery, Orthopedics University Hospital Frankfurt, Goethe University, Frankfurt, Germany; 3 Katharina-Schroth-Clinic, 55566 Bad Sobernheim, Germany

**Keywords:** Brace, Schroth therapy, Adolescent idiopathic scoliosis, Plantar pressure distribution, Gait symmetry

## Abstract

**Background:**

Positive effects of brace treatments in adolescent idiopathic scoliosis patients on gait were proven.

**Aim:**

Therefore, this study examined whether the influence of brace therapy in combination with Schroth therapy influencing the plantar pressure distribution, pre and post intensive rehabilitative inpatient treatment.

**Design:**

Prospective cohort study, longitudinal.

**Setting:**

Scoliosis rehabilitation clinic “Asklepios Katharina-Schroth-Klinik” (Bad Sobernheim, Germany).

**Population:**

Twenty (14f/6m) patients (12–16 years) had a medically diagnosed moderate idiopathic scoliosis (Cobb angle 20–50°, Median 30°) and an indication for combined brace and Schroth therapy with an inpatient stay (4 weeks) at the Asklepios Katharina Schroth Clinic (Germany).

**Methods:**

At the beginning (T1) and at the end of the stay (T2), the plantar pressure distribution with (A) and without wearing a brace (B) was recorded (walking distance 10 m).

**Results:**

No significant differences between the left and right foot were found at baseline (T1). The T1 - T2 comparison of one foot revealed significant differences (*p* ≤ 0.05 − 0.001, respectively) for (A): mean pressure right midfoot, loaded area total left foot, left midfoot, left inner ball of foot, right midfoot, impulse total right foot, right midfoot and for (B): mean pressure right midfoot, right outer ball of foot, loaded area total right foot, right heel, right midfoot, impulse right heel, right midfoot, right outer ball of foot.

**Conclusions:**

A combined brace and Schroth therapy maintains the initial symmetrical plantar pressure distribution over the duration of four weeks since the significant differences fall within the range of measurement error.

**Clinical rehabilitation impact:**

The insole measuring system can be used to objectively support therapeutic gait training as part of rehabilitation and to assess insole fitting based on foot shape. Due to its convenient handling and rapid data acquisition, it may be a suitable method for interim or follow-up diagnostics in the treatment of idiopathic scoliosis.

## Background

The gait pattern of adolescents with idiopathic scoliosis (AIS) is characterized by uneven translation and twisting [1]. Here, the pelvic position of the ipsilateral or contralateral half of the pelvis drops more noticeably when walking due to the curvature of the spine [2–5]. Thoracic curves of scoliosis for example may cause pelvic drop because they have sagittal profile of flat back and consequently this result in a ventral pelvic tilt to allow lordosis of the lumbar spine, while lumbar or thoracolumbar curves cause hyperkyphosis. In the case of main thoracic curvature, the trunk is still positioned in the center of the pelvis in the coronal plane [6]. With a main lumbar curve, however, the trunk deviates significantly to the convex side of the curve. In addition, a Cobb angle-dependent movement of the thorax in the sagittal plane can be observed [7]. However, the results could only be proven with special consideration of the individual curvatures, whereby it is assumed that the change in postural control also depends on the type of the curvature [6, 7]. According to radiographic imaging in two planes, asymmetry in the pelvis persists from the onset of the disease until the scoliosis progresses [8]. There is also a one-sided load on the inner edge of the foot, which is combined with a valgus upper ankle and knee. In addition, the imbalance is associated with an increased load on one leg and a disturbed sequence of body rotation and flexion [1]. The resulting deviation of the body’s plumb line leads to a lateral tilt of the trunk and a shift of the body’s center of gravity which can lead, for example, to an increased load on one leg and a disturbed course of body rotation [1]. The effects of scoliosis on posture and walking behavior indicate that scoliosis also has an effect on gait patterns, which can be detected by means of plantar pressure distribution, among other things. Here, Syczewska et al. [9] demonstrated the gait pattern dependence on the degree of curvature of the spine in thoracolumbar scoliosis and the presence of pelvic deformity. This imbalance could be closely related to the disproportion of the trunk musculature [8, 10] and thus the influence of gait. Consequently, deformities of the spine are often accompanied by pelvic obliquity and rotation, flat feet, high arched feet and other biomechanical imbalances of the lower limbs. Most often, unilateral loading on the same leg side as the accompanying scoliosis, so the intent is to reduce the risk of unilateral weight-bearing. Out of perpendicular, joint abrasion, like hip or knee, might increase.

In addition, da Silveira et al. [11] investigated the short- and long-term effects of brace use on the spine, balance and gait in adolescents with idiopathic scoliosis. They found improvements to the antero-posterior and medio-lateral body balance and in the analysis of the plantar pressure distribution a reduction in forefoot contact area, peak pressure and maximum force on the forefoot and rearfoot (medial and lateral) and peak pressure on the midfoot with immediate and long-term use of the brace (in combination with specific exercises).

A brace based on the three-point correction principle is intended to counteract the lateral torso inclination caused by the scoliosis from a curvature of the thoracic or lumbar spine of 20° according to Cobb [2] (medical therapy recommendation in Germany) [12, 13]. These recommendations for Germany [14] state that a brace should be worn if the Cobb angle is > 20°. Here, the main goal of orthotic treatment in adolescents with idiopathic scoliosis is to control the curve progression in those cases who are most at risk (curves 25°–45° and Risser sign 0–2). The brace is intended to normalize the body’s center of gravity and also to affect indirectly the gait pattern [12, 13, 15]. 

Mahaudens et al. [15] demonstrated that within six months of treatment, brace therapy brought the range of motion of the shoulder, pelvis, hip and ankle in 13 adolescent female idiopathic scoliosis patients (of mixed curvature type) closer to the more physiologic values of a control group. A decrease in the range of motion of the pelvis and hip by approximately 1–2° was found by Wong et al. [16] through brace therapy using rigid orthoses of 21 adolescent females (10–14 years old) over the course of one year. Similarly, Paolucci et al. [12] indicated an improvement in postural control (reduction in the body’s center of gravity sway and a reduction in extremity loading) by wearing a Cheneau brace compared with a control group in 13 patients (11f/2m) with an average age of 13.3 years. However, this was accompanied by a reduction in locomotion speed and stride frequency. The authors suggested that the brace counteracted the mechanisms of a faster gait. Gur et al. [13] demonstrated reduced fluctuations and improved adaptation during the raising and lowering of the toes as well as improved balance during standing, especially with the eyes closed, when comparing measurements made without wearing a brace with those in which a brace had already been worn for 30 min.

In addition to the therapeutic use of the brace for the treatment of scoliosis, movement-related (conservative) therapies are recommended for patients with severely pronounced scoliosis diagnosed by e.g. using the Cobb angle. The objective of conservative scoliosis therapies is to reduce the progression of scoliosis and to achieve a balanced force load on both legs. Enhanced outcomes can be attained when patients exhibit sagittal perpendicularity, alongside superior horizontal alignment of the shoulders and pelvis.

Here, the Katharina Schroth therapy [17, 18] is an effective form of conservative treatment for scoliosis. It is composed of various elements of physiotherapy in combination with breathing exercises and is based on the possible controllability of breathing [19]. These exercises are taught under supervision with the aim of slowing down the progression of scoliosis development and also maintaining a high quality of life as unrestricted as possible [19, 20]. 

Schreiber et al. [21] were able to demonstrate a significantly reduced curvature of the spine by 1.2° in a six-month course in 25 patients between 10 and 18 years of age, having spinal curves of 10–45° and a Risser grade of 0–5 by combining Schroth therapy with standard therapy. However, the major curvature worsened by 2.3° in a control group of 25 patients. Furthermore, Kim et al. [22] and Kwan et al. [23] concurred in their studies that a significant reduction of the Cobb angle could be achieved by using Schroth therapy. These results were confirmed by Hedayati et al. [24] revealing that combining shorter interval brace adjustments (twice per week) with group exercise increases patient satisfaction and reduces scoliosis Cobb angles.

Measurement methods used to date to monitor the progress of scoliosis (therapy) are, for example, electromyography (EMG) for muscle activity, motion capture systems for analyzing the gait pattern to obtain information on the axial position of the lower limb (hip, knee) or the rotational position of the feet or force plates for body balance. With these techniques, however, it must be considered that they are very cost-, time- and space-intensive and, therefore, tend to be the methods that are primarily used in a biomechanical laboratory and not in a rehabilitation clinic for the metrological support for medical progress controls or the diagnoses of idiopathic scoliosis patients. So far, the plantar pressure distribution has been used to evaluate the effects of orthotic insoles. Li et al. [25] improved human biomechanics and compensation in AIS patients within 2 months, especially foot dynamics during walking, by using orthotic insoles. The insoles improved the structural and functional abnormal biomechanics of plantar pressure (improved balance of force distribution) and thus influence gait and posture by changing the pressure distribution.

Consequently, the plantar pressure distribution is a measurement method that is suitable for the diagnostic support of gait training [11] and examination of the gait pattern in the therapy of idiopathic scoliosis patients in everyday clinical practice. The resulting parameters of the plantar pressure distribution can be used for statements concerning symmetrical pressure distribution between the left and right foot in total and for each foot zone (i.e. toe, bale, midfoot) as well as the rolling behavior or shape of the foot (e.g. high-arched foot, flat foot).

The plantar pressure distribution can be ideally recorded with the inner shoe measurement system. Over a walking distance of at least 12 m several consecutive step cycles of the left and right foot were recorded without a long preparation time.

Therefore, the aim of the present study was to assess the plantar pressure distribution of adolescents during a combined Schroth and brace therapy in the course of a four-week inpatient therapy. Since the question of plantar pressure distribution has not yet been investigated, a pilot study was undertaken. Thus, the questions to be clarified were to what extent the influences of the brace had on affecting the plantar pressure distribution and whether a harmonization of locomotion can be observed. Finally, it was established whether the inner shoe measuring system is suitable as a technical measuring support for the medical progress controls in idiopathic scoliosis patients as part of the daily routine of the clinic and whether meaningful findings can be obtained to complete the medical diagnosis. The hypotheses to be tested are:

1. Wearing the brace balances the mean pressure of the plantar pressure distribution during the rehabilitation stay.
2. The percentage ratio of deceleration and acceleration does not change during the rehabilitation stay.

## Materials and methods

### Participants

Twenty (14f/6m) adolescents aged 12–16 years (14.22 ± 1.35 years) participated in this prospective clinical study. All participants were first admitted to the Katharina-Schroth Klinik in Bad Sobernheim (Germany) as inpatients with a physician-diagnosed idiopathic scoliosis. The entire affected population has a Cobb angle of between 20–50° in their condition, thus, a combined brace and physiotherapy is indicated. The curvature types of the participants consisted of a thoracic, thoracolumbar or lumbar main localization.

Included in the study were patients with an inpatient stay of at least four weeks, aged 12 to 16 years, and who had physician-certified idiopathic scoliosis and were prescribed combined conservative brace and Schroth therapy. Prior experience of using Schroth therapy was available in the outpatient setting to varying degrees. Each patient already had a custom-made, fitted Cheneau brace [18, 26] in use before the start of the inpatient stay. However, this is a prerequisite for being allowed to undergo this rehabilitation stay. Nevertheless, the patients were all newly fitted with their brace and had received it a maximum of two weeks before the start of their first rehabilitation stay. In contrast, patients with neuropathic, congenital or syndromic scoliosis were excluded, as were those who had had surgery due to scoliosis and acute injury. In addition, voluntary participation and written informed consent from the patient’s legal guardians were required and obtained prior to the study.

Sample size was based on an effect strength of Rosenthals´*r* = 0.5, a power of 85% and an alpha of 5%, resulting in a total of 20 participants required (no dropouts were accounted for the sample size estimation). .

The study received ethics approval from the medical faculty of the Goethe University Frankfurt, Germany (No. 426/16). Informed consent was obtained from all participants and/or their legal guardian(s). All experimental protocols were approved by a named institutional and/or licensing committee. All methods were carried out in accordance with relevant guidelines and regulations.

### Plantar pressure distribution

**Fig. 1:**
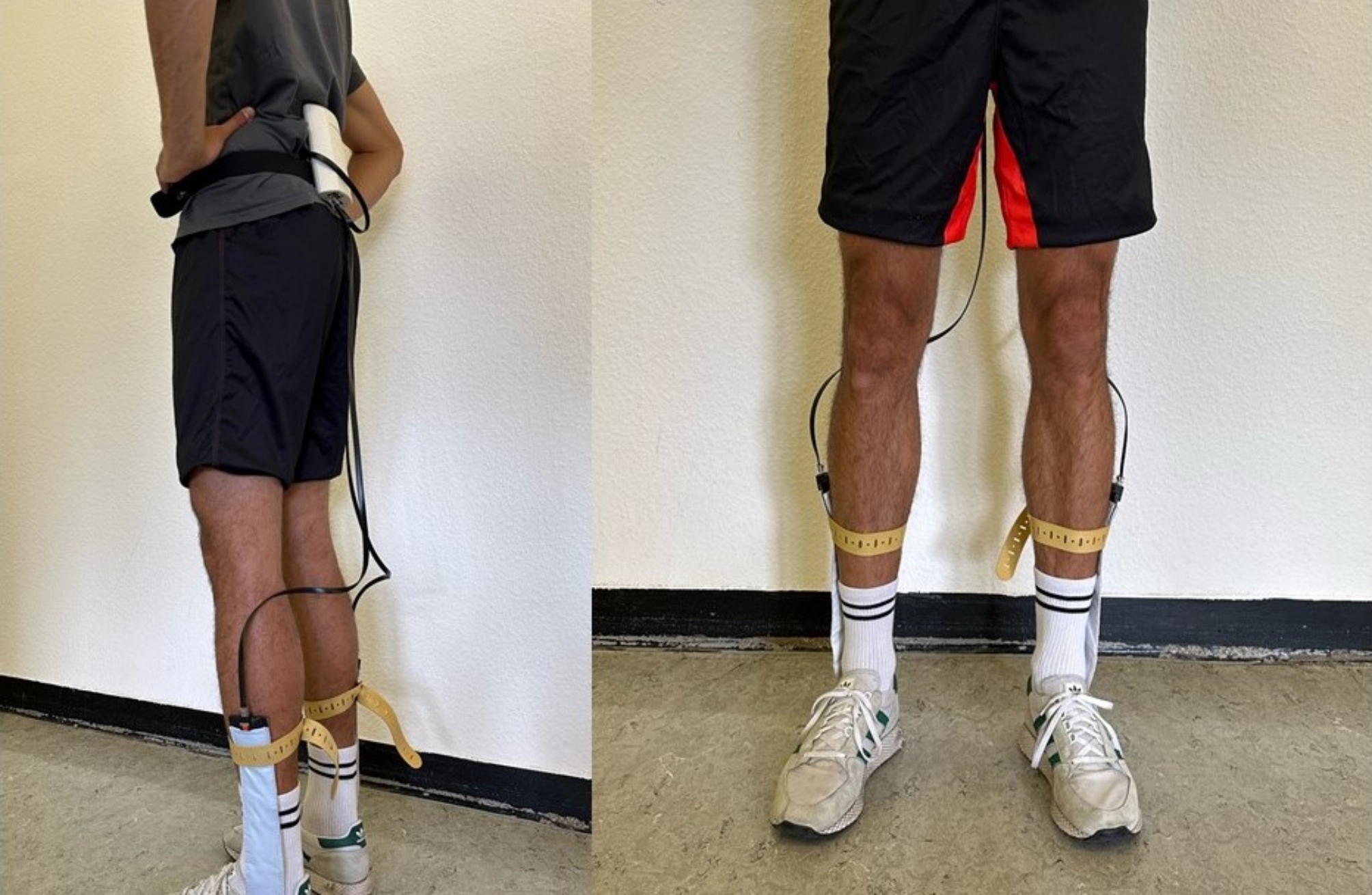
Illustration of the system to measure plantar pressure distribution

The GP MobilData Funk measurement system from GeBioM mbH (Münster, Germany) was used to determine the plantar pressure distribution during the study (Fig. 1). In this system, flexible insoles are used, corresponding to shoe sizes 36–46, which contain over 40–64 resistive sensors per sole size. The special multiplexing method interrogates each sensor at a sampling rate of 200 Hz with a measurement error of ± 5% according to the manufacturer’s specifications. For data acquisition, the sole of the foot is divided into seven different zones (heel (H), midfoot (M), inner ball (BI), medial ball (BM), outer ball (BO), toe (T) and the total foot (TF)). Five different parameters are recorded: the average pressure, the loaded area, the impulse, percentage ratio between deceleration and acceleration and the contact time.

### Examination procedure

Data collection took place at the beginning and end of the patient’s inpatient stay. For this purpose, measurement insoles corresponding to the patient’s own shoe size were placed in the patient’s own sports shoes (shoe size from 36 to 46). After selecting the correct size of the measuring insole, the insole was inserted into the shoe and checked to ensure that it neither displaced nor formed wrinkles when the shoe was put on. Test measurements were taken beforehand which were not included in the statistical evaluation. Each participant walked a total distance of 30 m (3 repetitions of 10 m each) at a self-selected constant speed. While walking the distance, the data of the left and the right foot were recorded. To prevent the measurements from influencing each other, there was an interruption of about 30 s between each data recording.

For the measurements themselves, there were two different measurement conditions: with and without wearing the individual’s brace. The mean values were calculated from the individual’s measurements, and from these the statistical evaluations were based.

### Statistical evaluation procedures

The software program BiAS (version 11.03, Epsilon Verlag, Darmstadt, Germany) was used for the calculations.

Firstly, all data were tested for normal distribution using the Kolmogorov-Smirnov test with Lilliefors correction. If the *p*-value is > 5%, a normal distribution is assumed. For normally distributed data, the T-test was used for dependent samples or, for non-parametric data, the Wilcoxon matched pairs test was used. With regard to gait symmetry, the differences between the left and right foot pre and post the inpatient stay were compared in each case. All *p*-values were subsequently subjected to a Bonferroni-Holm correction. The effect strength serves to evaluate the correlation coefficient r according to Rosenthal and is defined as follows: Rosenthal: 0.1 ‘small effect’, 0.3 ‘medium effect’, 0.5 ‘large effect’. The significance level was set at 5%.

Medians and 1st and 3rd quartiles were used for the descriptive presentation of the data in the graphs, as half of the data was not normally distributed.

## Results

All demographic and clinical characteristics of the 20 participants are listed in Table 1.

**Table 1:** Demographic and clinical characteristics of the subjects

	Age (years)	Sex	Body height (cm)	Body weight (kg)	Body-Mass-Index (kg/m²)	Cobb-angle thoracic (°)	Cobb-angle Lumbar (°)	Localization scoliosis
Number		14f 6 m						15 thoracic right/ lumbar left (double-arched) 4 thoracolumbar right 1 thoracic right
Mean	14.22		164.22	54.73	20.12	30.40	28.39	
Standard Deviation	1.35		7.70	12.19	3.05	10.75	8.39	
Median	14		164.5	50.30	19.60	34.00	30.00	
1./ 3. quartile	13/ 15		158.00/ 168.00	46.65/ 61.35	17.81/ 21.05	20.00/ 40.00	15.00/ 34.50	

### Average pressure

The comparison of with and without wearing a brace, at the beginning and at the end of the inpatient stay, showed no significant differences after Bonferroni-Holm correction (*p* ≥ 0.05) for the plantar pressure distribution of either the left or right foot.

The comparison pre and post therapy without wearing a brace showed a significant value with *p* = 0.01 (r: 0.41) in the area of the right midfoot, with median values pre therapy of 2.98 N/m² and post therapy of 2.58 N/m².

**Fig. 2:**
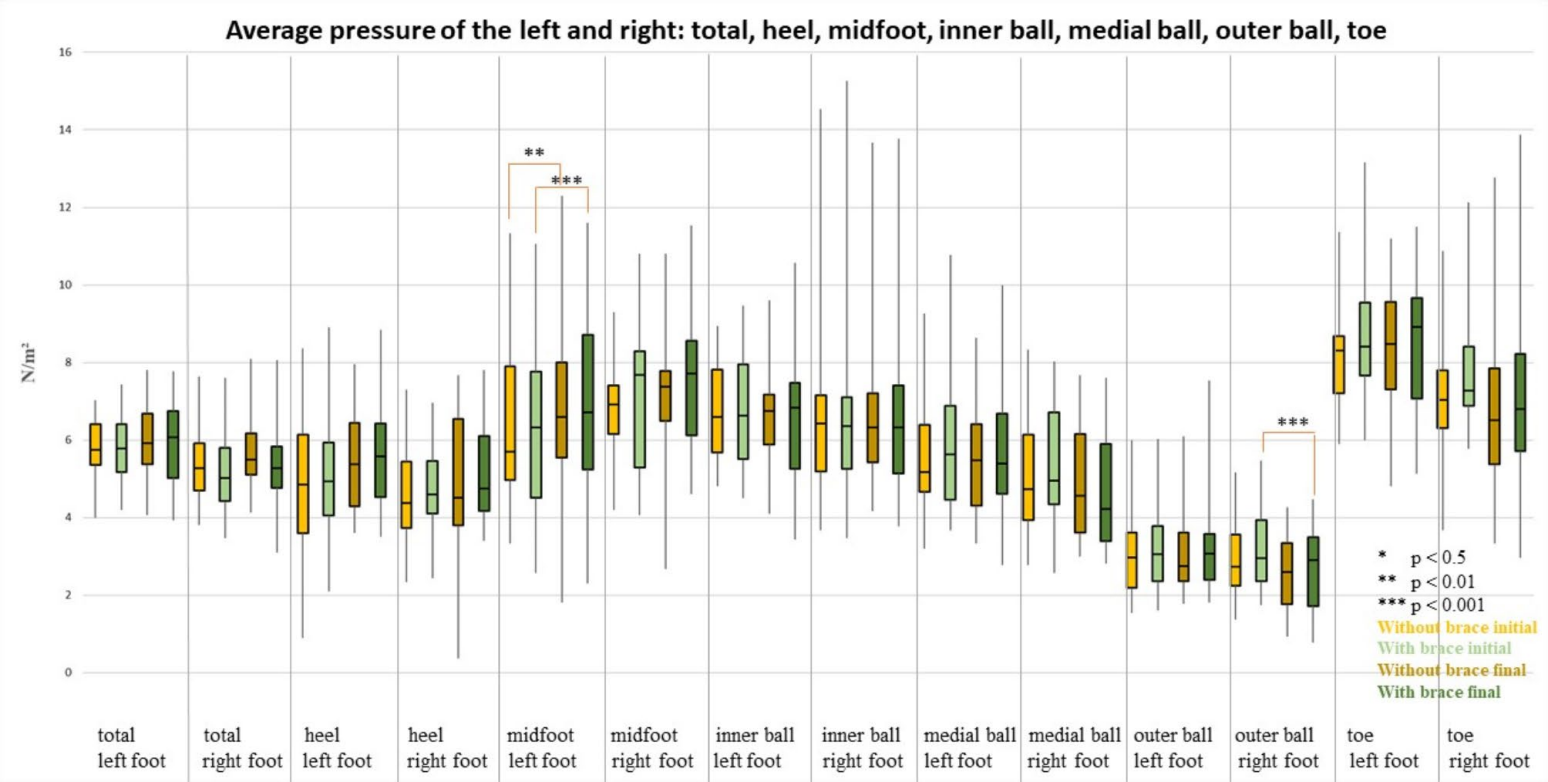
Representation of the average pressure (N/cm²) (median, 1. and 3. Quartile) of all participants with and without wearing a brace at the beginning and end of the inpatient stay. The colour coding is as follows: light yellow = without brace at the beginning of the inpatient stay, light green = with brace at the beginning of the inpatient stay; dark yellow = without brace at the end of the inpatient stay; dark green = with brace at the end of the inpatient stay. Significant differences are marked with asterisks: * *p* < 0.5, ** *p* < 0.01, *** *p* < 0.001

When wearing a brace, significant *p*-values of *p* = 0.001 (r: 0.46 and 0.48 respectively) were found in the right metatarsal and right outer ball of the foot (median values; right metatarsal: pre 3.16 N/m², post 2.70 N/m²; right outer ball of the foot: pre 5.40 N/m², post 4.78 N/m²). No significant differences could be determined for the remaining parameters (Fig. 2).

### Loaded area

When the measurements from wearing or without wearing a brace, taken pre and post treatment, were compared; it was found that all zones adopted almost identical values and were not significantly different.

The Bonferroni-Holm corrected *p*-values of the measurements pre and post treatment without orthosis revealed significant values in the entire left foot (*p* = 0.02, median pre 18250 mm², median post 18229.17 mm², r: 0.37), the left midfoot (*p* = 0.01, m median ean pre 4700 mm², median post 4683.33 mm², r: 0.39), the inner left ball of the foot (*p* = 0.01, median pre and post 2150 mm², r: 0.60), and the right midfoot (*p* = 0.001, median pre 4500 mm², median post 4495.83 mm², r: 0.56).

**Fig. 3:**
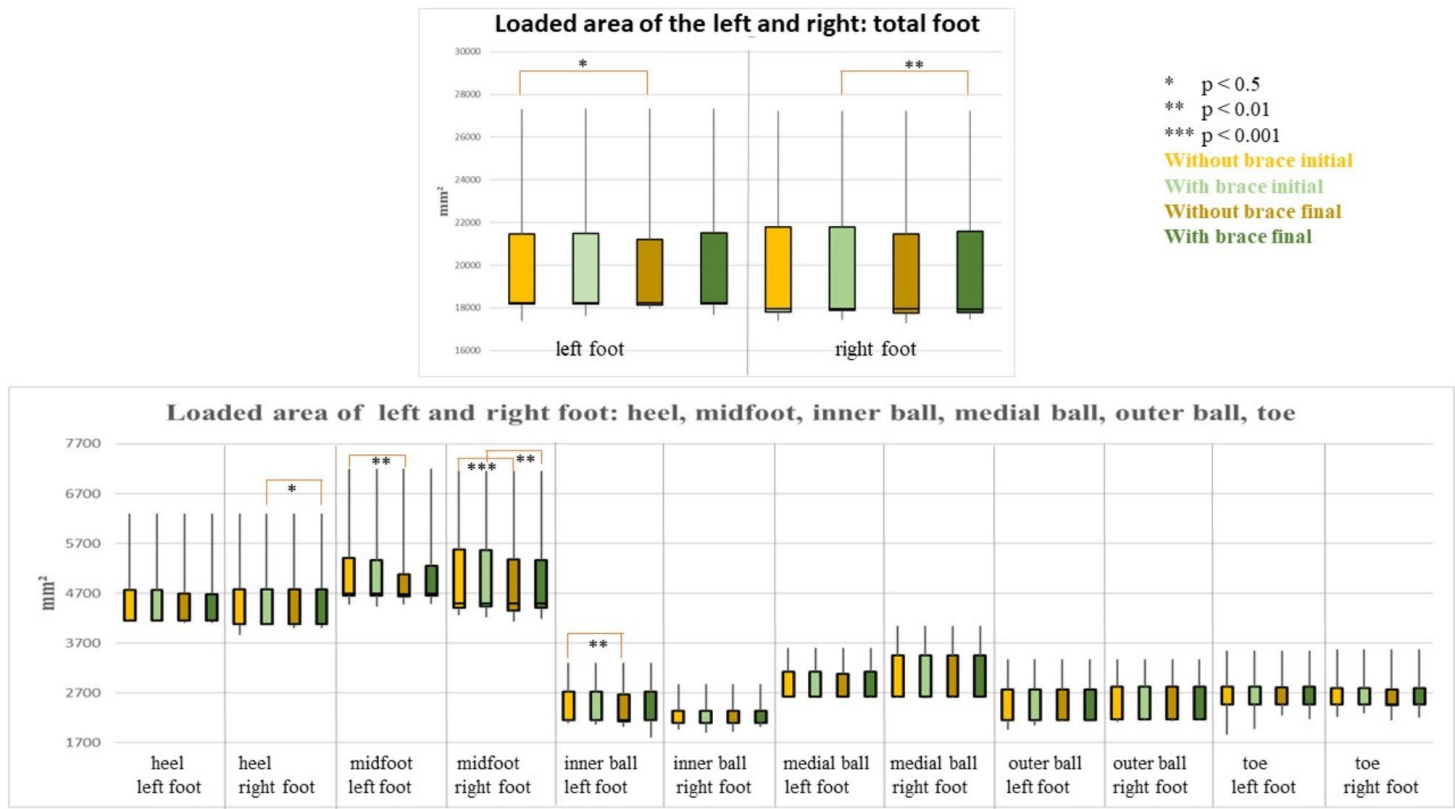
Representation of the loaded area (mm²) (median, 1. and 3. Quartile) of all participants with and without wearing a brace at the beginning and end of the inpatient stay of the total foot (top of the figure) and the other foot areas (bottom of the figure). The colour coding is as follows: light yellow = without brace at the beginning of the inpatient stay, light green = with brace at the beginning of the inpatient stay; dark yellow = without brace at the end of the inpatient stay; dark green = with brace at the end of the inpatient stay. Significant differences are marked with asterisks: * *p* < 0.5, ** *p* < 0.01, *** *p* < 0.001

Finally, a comparison of the measurements pre and post therapy when wearing a brace was made. A significant difference was found for the entire right foot (*p* = 0.01, median pre 17950.00 mm², median post 17937.50 mm², r: 0.44), the right heel (*p* = 0.03, median pre and post 4075 mm², r: 0.60), and the right midfoot (*p* = 0.01, median pre 4,500 mm², median post 4,495.83 mm², r: 0.44). No significant differences could be determined for the remaining comparisons (Fig. 3).

### Impulse

The comparison of the impulse data from with and without wearing a brace, pre and post inpatient stay, showed no significance for either the left or right foot.

The comparison of the data without wearing a brace pre and post stay revealed significant differences for the entire right foot (*p* = 0.001, median pre 316.93 Ns, median post 244.65 Ns, r: 0.45) and the right midfoot (*p* = 0.001, median pre 37.27 Ns, median post 20.46 Ns, r: 0.55).

**Fig. 4:**
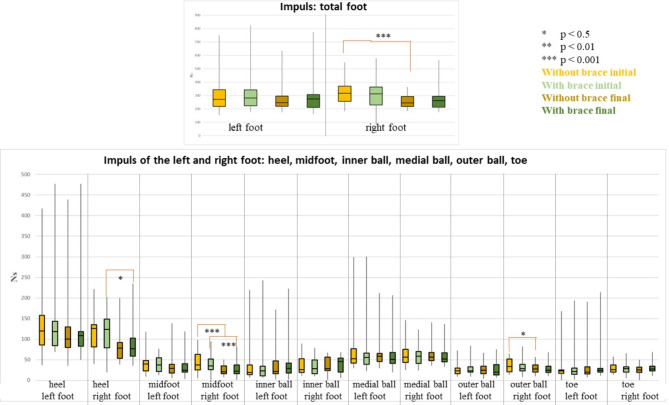
Representation of the loaded area (NS) (median, 1. and 3. Quartile) of all participants with and without wearing a brace at the beginning and end of the inpatient stay of the total foot (top of the figure) and the other foot areas (bottom of the figure). The colour coding is as follows: light yellow = without brace at the beginning of the inpatient stay, light green = with brace at the beginning of the inpatient stay; dark yellow = without brace at the end of the inpatient stay; dark green = with brace at the end of the inpatient stay. Significant differences are marked with asterisks: * *p* < 0.5, ** *p* < 0.01, *** *p* < 0.001

When comparing the data for wearing a brace pre and post therapy, significant *p*-values could be found in the right heel (*p* = 0.02, median pre 123.38 Ns, median post 76.56 Ns, r: 0.37), the right midfoot (*p* = 0.001, median pre 35.15 Ns, median post 22.27 Ns, r: 0.43) and the right outer ball of the foot (*p* = 0.02, median pre 28.47 Ns, median post 24.75 Ns, r: 0.38). The remaining results did not show any significant differences (Fig. 4).

### Force rate

**Fig. 5:**
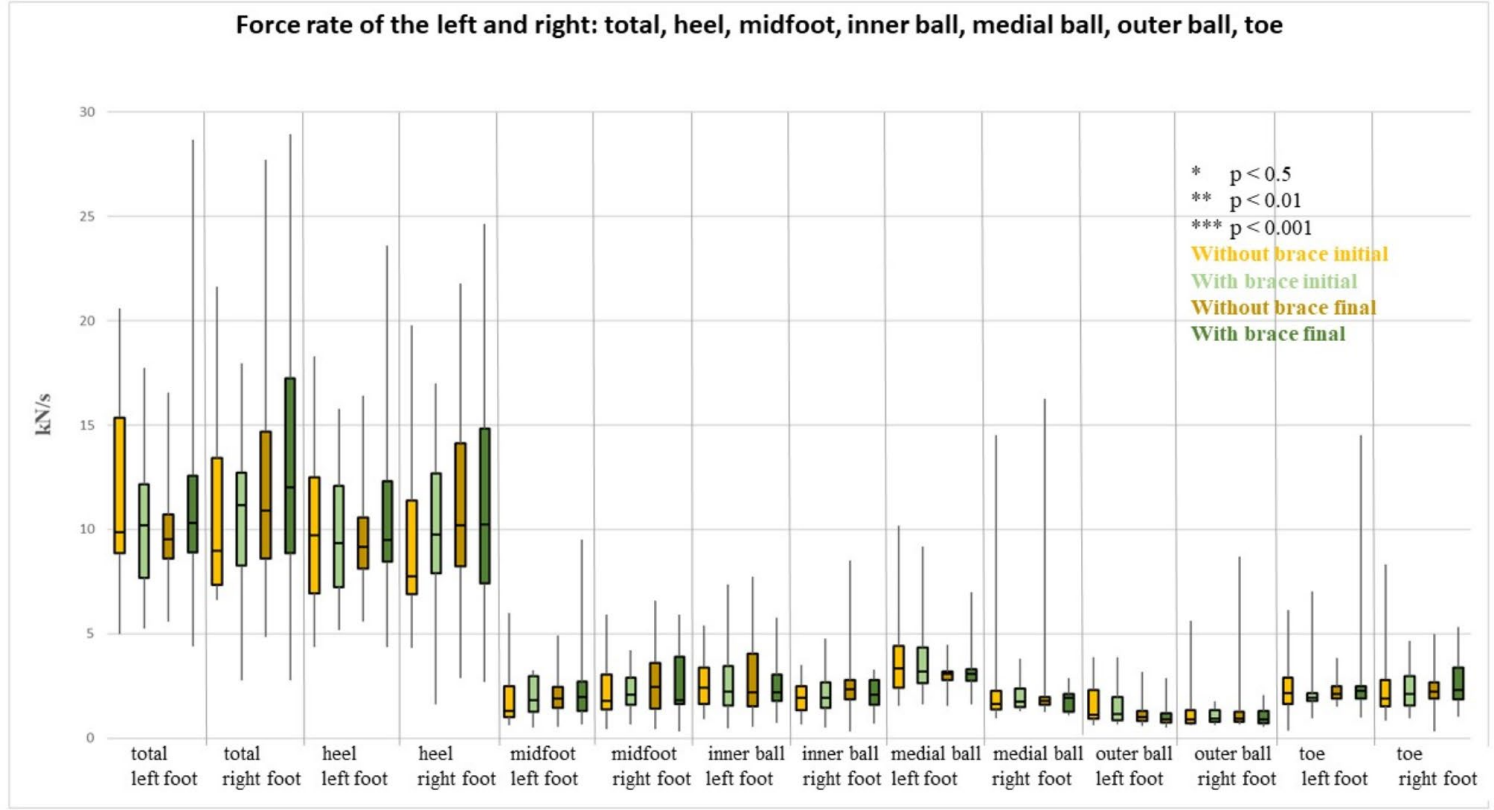
Representation of the force rate (kN/s) (median, 1. and 3. Quartile) of all participants with and without wearing a brace at the beginning and end of the inpatient stay. The colour coding is as follows: light yellow = without brace at the beginning of the inpatient stay, light green = with brace at the beginning of the inpatient stay; dark yellow = without brace at the end of the inpatient stay; dark green = with brace at the end of the inpatient stay. Significant differences are marked with asterisks: * *p* < 0.5, ** *p* < 0.01, *** *p* < 0.001

All comparisons were not significant with regard to the force rate; this included the intra-(without) brace pre-post comparison as well as the stay comparison, at the beginning and at the end, of the with vs. without wearing a brace (Fig. 5).

### Percentage ratio of the deceleration and acceleration process

**Fig. 6:**
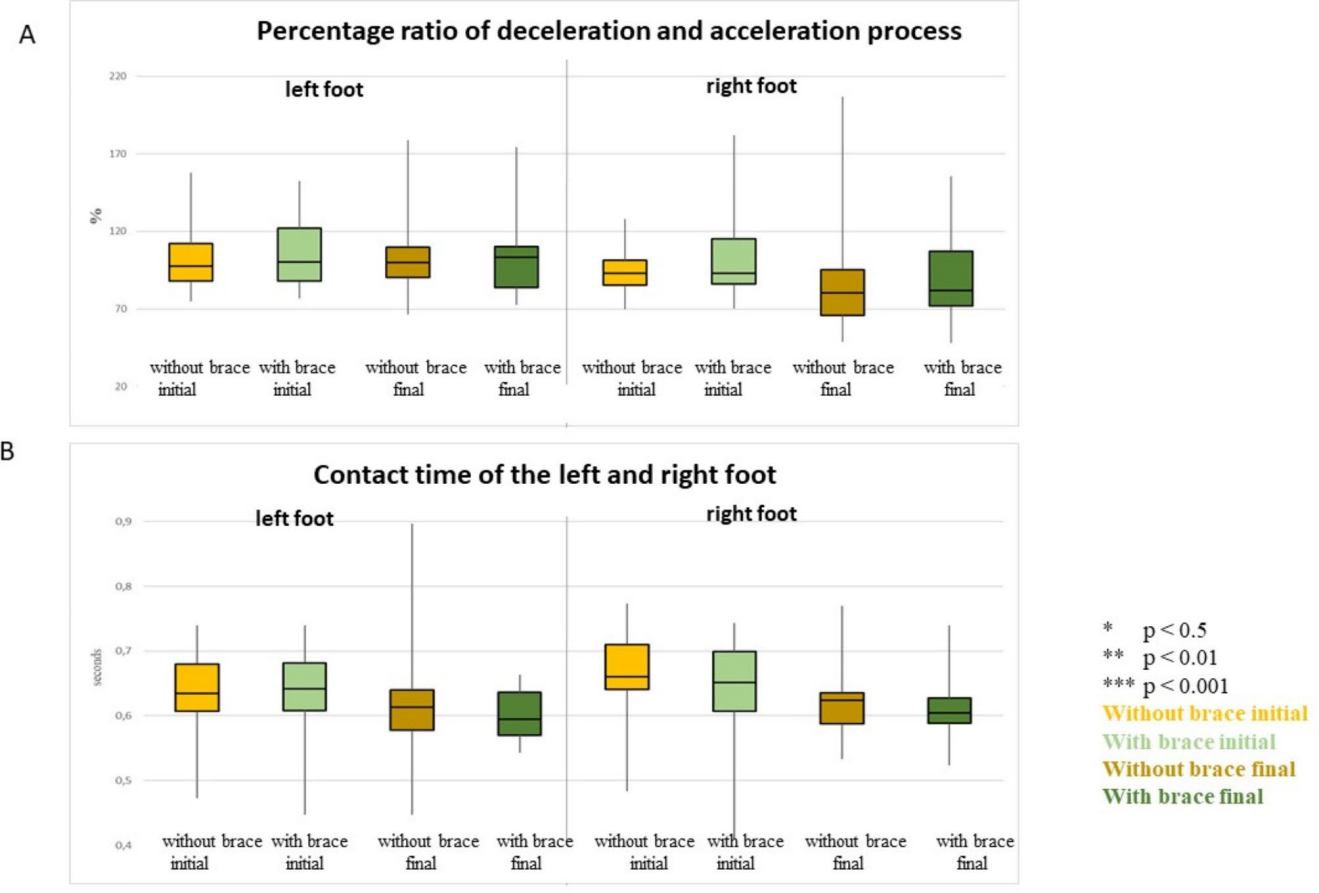
**A** shows the median, 1st and 3rd quartiles of the percentage ratio of deceleration and acceleration of the left and right foot, whereas **B** shows the contact time of the right and left foot of all participants with and without wearing a brace at the beginning and end of the inpatient stay. The colour coding is as follows: light yellow = without brace at the beginning of the inpatient stay, light green = with brace at the beginning of the inpatient stay; dark yellow = without brace at the end of the inpatient stay; dark green = with brace at the end of the inpatient stay. Significant differences are marked with asterisks: * *p* < 0.5, ** *p* < 0.01, *** *p* < 0.001

The percentage ratio of the deceleration and acceleration process indicated a value approximating 100% at the beginning of the measurements; this was confirmed for all conditions and did not change beyond the known measurement inaccuracy of 5% (Fig. 6A). Results showed no significant differences between initial and final testing.

### Contact time

There were no significant difference between initial and final testing for all comparisons of the contact time (Table 2; Fig. 6), thus, the walking speed was found to be the same in each measurement.

**Table 2:** Representation of the medians and 1st and 3rd quartiles of contact time with and without wearing a brace at the beginning and end of the inpatients’ rehabilitation stay (in sec.)

	Beginning of the inpatients’ rehabilitation stay						End of the inpatients’ rehabilitation stay						
	Without brace			With brace			Without brace				With brace		
	Median	*1st Quartile*	*3rd Quartile*	Median	*1st Quartile*	*3rd Quartile*	Median	*1st Quartile*	*3rd Quartile*	Median		*1st Quartile*	*3rd Quartile*
Total	0.66	0.64	0.71	0.65	0.60	0.70	0.62	0.58	0.64	0.60		0.58	0.64

### Gait symmetry

Only for the impulse could a significant *p*-value be detected after Bonferroni-Holm correction in the midfoot area (*p* = ≤ 0.001, pre 1.61 Ns, post 16.67 Ns, r: 0.45) with and without wearing a brace. All other foot zones were not significant. The same was true for all zones in the other parameters.

## Discussion

Since there are no comparative analyses available, to date, regarding the effects of combined Schroth and brace therapy during a four-week inpatient rehabilitation on plantar pressure distribution, the present study can be considered as a pilot study.

The results of this investigation showed isolated significant differences in the pre-post comparison of the inpatient rehabilitation stay, with or without wearing a brace, per left/right foot as well as in the comparison of the condition with vs. without wearing a brace.

Most of these significant differences showed a medium effect size. However, the measurement error of the system of 5% must be taken into account when evaluating the findings. The consideration of this percentage difference in the mean values or median values reduces the clinical importance (predominantly medium effect size) of the significant differences.

Rather, it can be seen from the measurements that the plantar pressure distribution was already essentially balanced at the beginning of the data collection, since the recorded differences in mean pressure and force rate in the respective foot zones between the left and right foot were, for the most part, very small and did not change significantly even after four weeks of therapy, i.e., neither an improvement nor a worsening had occurred. Furthermore, there was no change in the longitudinal and transverse arch, recognizable by the loaded area of the measurement sensors in the midfoot and forefoot areas, respectively. The impulse with slight temporal additional loading of the forefoot also remained mostly constant over all the measurements. Another aspect that brings the gait pattern (which was already balanced at the beginning) to the fore is the percentage ratio of deceleration and acceleration; this already conveyed an even ratio between the heel strike and forefoot push-off at the start of the measurements (almost 100%). Since the contact time remained constant for all measurements, a speed effect can be excluded.

Therefore, hypothesis 1 has to be rejected and hypothesis 2 has to be verified. Following these results, the question arises of why the pelvic position or pelvic movements and postural control can be positively influenced by therapy [12, 13, 15] even though the plantar rolling behavior is already balanced at the beginning of the stay and remains constant during the inpatient’s stay.

In theory, it can be assumed that a human body, when affected by a deformity, attempts to restore an “anatomical-physiological” balance through neuromuscular compensation processes [27]. The pelvis connects the spine with the lower extremity and, as the basis of motion transmission, can contribute to a significant part of the expression of the gait pattern. Opposing curves are formed in idiopathic scoliosis in order to maintain the balance of the body, while biomechanical as well as neuromuscular, compensatory processes also counteract the deformity. These neurophysiological processes seem to compensate so well that, although positive influences of the brace can be seen in the trunk area [15], the plantar pressure distribution can be balanced from the beginning. Accordingly, this confirms the positive consequences of these compensatory mechanisms. A prior influence as well as a habituation effect from the brace can be ruled out because the patients of the present study, on the one hand, had not yet had an inpatient stay and, on the other hand, had only a few physiotherapeutic hours prior to the study. The patient-dependent individual, variable Schroth therapy [28], which was intensively performed during the stay, may have positively supported this effect.

The influence of a brace on plantar pressure distribution in patients with AIS has only been investigated by Li et al. [25] to date. They demonstrated an asymmetric plantar pressure distribution in AIS patients regardless of randomized group allocation (brace vs. brace and insoles). All patients had a moderate Cobb angle (approx. 33°), similar to the patients in the present study. However, the localization of scoliosis in Li et al. [25] was not mentioned. They demonstrated that the plantar pressure center drift index in the AIS group was significantly higher (approximately 4°) than that in the healthy group. Additionally, they were able to show significant differences in the ratio of medial and lateral heel pressure (M/l) and total foot pressure, with an average ratio deviation between 0.03 and 0.16. Similarly, small significant differences in the median values were found in the present study pre and post brace therapy, although other parameters and assessment methods of the plantar pressure distribution were used. In addition, the patients in the present study had predominantly moderate thoracolumbar scoliosis, whereas the exact localization of the moderate Cobb angle in Lie et al. [25] is not published.

The present results are, thus, in contrast to other research, although other measurement methods were used in those studies, such as an optical motion detection system [15, 16] or a baropodometric pressure measurement platform [12, 13]. When analyzing the results of the previously listed studies [12, 13, 15, 16], it can be seen that in some cases only individual significant values were calculated, or that the quintessence was often inferred from tendencies. Here, however, the clinical relevance of the statements or significant results (in part, very small significant differences (e.g., as small as 1–2° by Wong et al. [16]) should be critically considered. However, the patient population in the present study is identical to that of the previously mentioned studies: adolescent idiopathic scoliosis patients with a similar average age of around 14.5 years [12, 13, 15, 16]. 

### Strength and limitations

So far, it was previously unclear whether and to what extent this combined therapy has an effect on plantar pressure distribution, with particular consideration given to harmonization, as data from the left and right foot are correlated. This information is important in the context of an accompanying gait training as part of the therapy, which to date has only been based on the clinical experience of the practitioner, and the additional information for a hypothetical insole fitting.

In Germany, the medical therapy indication for an adolescent with scoliosis is based on the S2 guideline for “Adolescent Idiopathic Scoliosis”. Medical indication is always based on an X-ray and the classification of the Cobb angle according to the S2 guideline. This guideline states that scoliosis between 20°-50° must be treated with brace and conservative therapy. Lesser scoliosis only receive conservative therapy, such as Schroth therapy. Scoliosis > 50° therefore has an indication for surgery. A comparison with participants groups in which only scoliosis with a Cobb angle of < 20° or > 50° are included would not be comparable due to different criteria. A control group is therefore not possible, according to the classification criteria. It would also be difficult to find adolescents who would voluntarily undergo daily Schroth therapy without medical indication or undergo the combination of brace and Schroth therapy. Also, no child with a Cobb angle < 20° would voluntarily wear a brace.

The study did not include a radiographic examination to assess leg length discrepancy, which might affect plantar pressure distribution. Additional X-rays are not ethically justifiable in the context of this therapy and are not treatment-induced. Generally, the clinical findings of an orthopedist or pediatrician are sufficient in Germany. During the preliminary examinations, a pediatrician and an orthopedist examined all patients at the clinic. However, this would have been noted in the patient’s file by the respective clinic physician, so that we did not consider this aspect further.

We only examined adolescents with moderate idiopathic scoliosis with combined brace and Schroth therapy who consequently had a Cobb angle between 20–50°. This is due to the fact that in Germany the medical therapy indication for an adolescent with scoliosis based on the S2 guideline for “Adolescent Idiopathic Scoliosis”. Medical indication is always based on an X-ray and the classification of the Cobb angle according to the S2 guideline [14]. This guideline states that scolioses between 20°-50° must be treated with brace and conservative therapy. Milder scolioses only receive conservative therapy, such as Schroth therapy. Scolioses > 50° therefore have an indication for surgery. A control group is therefore not possible, as the classification states. Also, the observation design without a control group is a limitation.

The inner shoe measuring system can reproduce data for the left/right foot in total and as well as for each zone (i.e. toe), the contact time of a foot/individual foot zones and the percentage relationship between the rolling and acceleration processes. However, no statement could be made as to whether the direction of rotation of the foot during walking changes as a result of the combined brace-Schroth therapy. Consequently, although the rolling behavior was found to be identical on both feet, the rotational positioning may have changed. This should be explored in further studies.

### Future research

The inpatient treatment lasted four weeks. Whether this treatment time is sufficient to observe significant changes in the plantar pressure distribution or the long-term effects of the therapy cannot be fully answered on the basis of the available data to date, as no comparative data from other studies exist. This period of rehabilitation time is specified by German pension insurance, which is legally prescribed for this therapy for adolescents. But these aspects should be the subject of future studies. Furthermore, it is not apparent from the current literature research whether the gait pattern of adolescents differs from that of adult scoliotic patients, and to what extent. Moreover, in this regard, it should be considered that divergent, asymmetric spinal loads could develop from the degree of the curvature and the localization of the scoliosis. This, in turn, could have a varying impact on the effect of the brace fitting. However, further studies should be performed on this to detect any possible divergence. It also remains to be investigated whether a brace-induced pelvic correction results in altered muscle activation of the foot muscles.

The aforementioned ideas for future investigations should incorporate additional measurement techniques alongside plantar pressure distribution to adequately address both the static and dynamic components of human body posture.

In addition to (combined) brace and Schroth therapy, gait training is a sub-area of rehabilitation.

This can be individually supported with orthotic insoles. One objective of insoles is to compensate for muscular imbalances and achieve a perpendicular posture. In this context, the question of shoe enhancement or compensating for a difference in leg length by means of an insole enhancement in order to distribute loads better also arises when providing insoles. During gait training, the correct gait pattern should be consciously learned (including rolling, inversion, eversion, pronation, supination). With the data from the plantar pressure distribution, the therapeutic gait school can be better coordinated. Furthermore, the shape of the foot (e.g. high-arched foot, flat foot) and the load distribution in individual foot zones can be recognized more precisely. The foot shape in particular provides an indication of whether and, if so, which foot orthosis should be indicated. However, whether the therapy combination had an effect on the rotational position of the feet cannot be investigated solely through plantar pressure distribution.

## Conclusion

In summary, this observational cohort study has shown that the plantar pressure distribution in adolescents with idiopathic scoliosis (median thoracic Cobb angle 30°) is already quite balanced between the right and left foot. In addition, the gait pattern is maintained over a period of four weeks of corset therapy in combination with Schroth therapy and does not deteriorate, although there is a certain brace-related influence on the pelvic position and posture. Due to its convenient handling and fast data acquisition, the insole measuring system may be a suitable method for interim or follow-up diagnostics in the treatment of idiopathic scoliosis. This is particularly the case when orthotic intervention is integrated into the therapy.

## Data Availability

All data is included within the manuscript.
